# Comparative Analysis of Ultrasound-Guided Pain Management Approaches for Sternotomy in Cardiac Surgeries—Transversus Thoracic Muscle Plane Block vs Pecto-Intercostal Fascial Block

**DOI:** 10.31486/toj.24.0052

**Published:** 2025

**Authors:** Hemant Vanjare, Chetana Prakash Deshmukh, Swapnil Kumar Barasker, Akheela Mohamed Kassim, Bipin Arya

**Affiliations:** ^1^ Department of Anaesthesia, Sri Aurobindo Medical College & PG Institute, Indore, Madhya Pradesh, India; ^2^ Department of Community Medicine, Sri Aurobindo Medical College & PG Institute, Indore, Madhya Pradesh, India; ^3^ Department of Anaesthesia, Jawaharlal Institute of Postgraduate Medical Education and Research, Pondicherry, Tamil Nadu, India

**Keywords:** *Cardiac surgical procedures*, *nerve block*, *pain management*, *pain–postoperative*, *ropivacaine*

## Abstract

**Background:**

Pain management after sternotomy in cardiac surgery is vital for recovery. Opioids are commonly used, but they carry risk. Central neuraxial techniques and nerve blocks are options for a multimodality approach. Fascial plane blocks such as the transversus thoracic muscle plane block (TTMPB) and the pecto-intercostal fascial block (PIFB) are a relatively new way to relieve pain, and their popularity has increased with the use of ultrasound for precise anatomic visualization. Because the effectiveness of both blocks is similar, we conducted this study to compare the pain management of the TTMPB and the PIFB after sternotomy in cardiac surgery.

**Methods:**

This randomized double-blind study included 118 patients who underwent cardiac surgery. In the TTMPB group (n=59), 20 mL of 0.2% ropivacaine was injected bilaterally using ultrasound assistance in the transversus thoracic plane. In the PIFB group (n=59), 20 mL of 0.2% ropivacaine was injected in the pecto-intercostal plane. Study outcomes were opioid consumption in the first 24 hours and pain scores at 0, 3, 6, 12, and 24 hours postoperatively.

**Results:**

Patient characteristics in the 2 groups were similar. Opioid consumption was similar in both groups (*P*=0.672), and we found no difference in pain scores between the 2 groups at any of the time intervals.

**Conclusion:**

The TTMPB and the PIFB were similarly effective in treating acute poststernotomy pain in our patient population.

## INTRODUCTION

Optimal pain management following sternotomy in cardiac surgery plays a crucial role in facilitating a swift return to regular activities.^1^ Sternotomy pain has the potential to lead to pulmonary issues, including inadequate secretion clearance, prolonged weaning from the ventilator, acute respiratory failure because of shallow breathing, and ineffective coughing. Effective pain management can mitigate these complications and thereby reduce postoperative morbidity and mortality.^2^ Although the use of opioid-based analgesia strategies is currently predominant after cardiac surgery, opioids are associated with adverse effects such as respiratory depression, nausea, vomiting, itching, urinary retention, and the risk of addiction.^3^

Thoracic epidural blocks have been shown to be effective in managing pain following cardiac surgeries.^4^ However, some anesthesiologists are hesitant to use these blocks because of concerns about spinal hematoma development from heparinization and hemodilution.^5^

The use of fascial plane blocks to treat pain began emerging in clinical practice in the early 2000s, with significant interest and research developing by 2016.^6^ The concurrent increase in the use of ultrasound for precise anatomic visualization prompted anesthesiologists to opt for regional anesthesia techniques.^7^ Fascial plane blocks such as the transversus thoracic muscle plane block (TTMPB) and the pecto-intercostal fascial block (PIFB) have a lower risk of bleeding compared to central neuraxial techniques or deep nerve blocks and are increasingly being integrated into multimodal analgesia protocols as an alternative strategy for pain management in cardiac surgery.^8^

**Figure 1.**
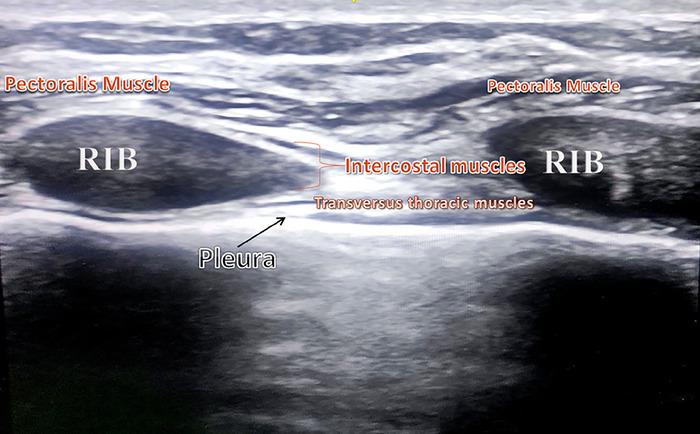
Ultrasound image of anatomic structures with the probe positioned parallel to the sternum at the level of the fourth intercostal space.

**Figure 2.**
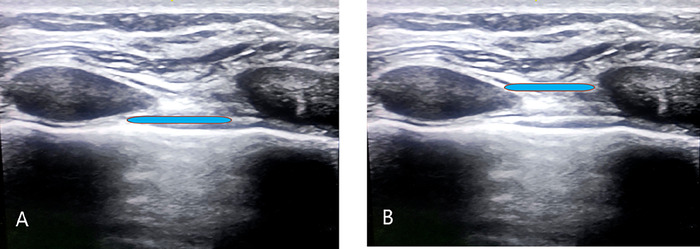
Ultrasound-guided local anesthetic placement for the (A) transversus thoracic muscle plane block and the (B) pecto-intercostal fascial block.

Most sternotomy pain results from tissue injury to the skin, subcutaneous tissues, bone, and cartilage; the sternum, ribs, and surrounding subcutaneous tissues are innervated by intercostal nerves arising from thoracic nerve roots, particularly T2 to T6.^9^ Ueshima and Kitamura published a report in 2015 suggesting that the TTMPB may block several anterior branches of the intercostal nerves (T2 to T6) that innervate the internal mammary region, including the sternum.^10^ For the TTMPB, local anesthetic is injected in the space between the internal intercostal and transversus thoracic muscles (Figures 1 and 2A), covering the anterior intercostal nerve branches from T2 to T6 and providing analgesia to the sternum region. de la Torre et al described a similar block, the PIFB, for breast surgeries in which local anesthetic is injected between the pectoralis major and external intercostal muscles (Figures 1 and 2B).^11^

Because injecting the local anesthetic between these 2 muscles blocks the same nerve roots, their efficacy should be comparable in terms of pain score. To date, several studies have been conducted on the TTMPB, but few studies compare the TTMPB with the PIFB.^12,13^ We designed our study based on the hypothesis that the perioperative analgesic effectiveness of the TTMPB and the PIFB in patients undergoing cardiac surgery via median sternotomy is comparable in terms of opioid consumption and pain scores.

## METHODS

Following approval from the institutional ethical committee (SAIMS/IEC/12/23) and registration with the central trial registry (CTRI/2024/01/061291), we conducted a prospective randomized double-blind trial in a tertiary care university teaching hospital from December 2023 to April 2024. Study procedures were conducted in accordance with the Declaration of Helsinki 2013. After the procedure and specific study intervention were explained, written informed consent was obtained from patients.

One hundred twenty-four patients planned for cardiac surgery through midline sternotomy were enrolled in the study. Patients with the following conditions were excluded: (1) not extubated during the first 24 hours, (2) undergoing a repeat cardiac surgical procedure, (3) history of coexisting coagulation disorders, (4) poor left ventricular ejection fraction (<35%) preoperatively, (5) neuromuscular problems, (6) infections at the injection site or systemically, (7) any known allergy to the local anesthetic used, (8) emergency surgery, (9) psychiatric illness, and (10) opioid dependency. Patients were randomized into 2 groups—the TTMPB group and the PIFB group—of 59 patients each through block randomization using computer-generated random numbers. The group allocation numbers were concealed in sealed opaque envelopes that were opened after patient enrollment. A sole proficient anesthesiologist with 5 years of expertise in ultrasound-guided regional anesthesia performed all the blocks; however, the anesthesiologist was not engaged in postprocedure observations. The anesthesiologist responsible for data collection was blinded to the study group.

### Procedures

All patients underwent a comprehensive preanesthetic assessment before surgery. Standard investigations were conducted following hospital protocol, and patients were instructed to fast for 8 hours prior to the surgical procedure. Before the day of surgery, patients were briefed on the study protocol and introduced to the numeric rating scale (NRS). In the preinduction room, intravenous midazolam (1 to 3 mg) was used as a premedication. After standard monitors were connected, fentanyl (5 to 6 μg/kg), propofol (2 mg/kg), and atracurium (0.5 mg/kg) were used to induce anesthesia, after which each patient was maintained with oxygen and air at a ratio of 1:1 with isoflurane on controlled mechanical ventilation. Heart rate and blood pressure were maintained within 20% of baseline levels. After endotracheal intubation, arterial and central venous catheters were inserted.

After induction, patients in the TTMPB group were positioned supine, and following proper sterilization, an L4-12t-RS linear probe of the Venue Go ultrasound system (GE HealthCare) was used to identify the anatomic plane between the internal intercostal and transversus thoracic muscles (Figures 1 and 2A). A Stimuplex A, 21-gauge, 4-inch needle (B. Braun Medical Inc) with a short bevel was inserted into the fourth intercostal space, extending to the sternum. The entire length of the needle and placement between the 2 muscles were visually confirmed. After a negative aspiration test, 1 mL of local anesthetic solution was administered. Achieving the TTMPB block involved injecting 20 mL of 0.2% ropivacaine, and the same procedure was repeated on the opposite side.

For patients in the PIFB group, a Stimuplex A, 21-gauge, 4-inch needle (B. Braun Medical Inc) was placed in the caudal-to-cephalad direction with an in-line technique. After needle placement was verified with ultrasound, 20 mL of 0.2% ropivacaine was injected in the space between the pectoralis major and external intercostal muscles (Figures 1 and 2B).

Following surgery, all patients were moved to the intensive care unit (ICU) for postoperative monitoring and care. Patients were extubated when they met the following criteria: alert and responsive, stable hemodynamically, no ongoing bleeding, warm extremities, and satisfactory arterial blood gas levels with fraction of inspired oxygen <50%.

### Outcome Measures and Assessment

The primary outcome measure was opioid consumption during the first 24 hours. Secondary outcome measures were NRS pain scores obtained during normal tidal volume breathing and during coughing at 0, 3, 6, 12, and 24 hours postextubation; time to first rescue analgesia (hours); time to extubation; duration of ICU stay (hours); and postoperative complications.

### Statistical Analysis

Data are presented as means ± standard deviations, medians and interquartile ranges, or frequencies and percentages as appropriate. Comparative analysis between study groups for numeric variables utilized the *t* test for independent samples. Changes in the NRS score over time within each group were assessed using repeated measures analysis of variance. Categorical data were compared using the chi-square test, with exact tests employed when expected frequencies were <5. A 2-sided *P* value <0.05 was deemed statistically significant. SPSS Statistics version 22 (IBM Corporation) for Microsoft Windows (Microsoft Corporation) was used to perform all statistical analyses.

## RESULTS

**Figure 3.**
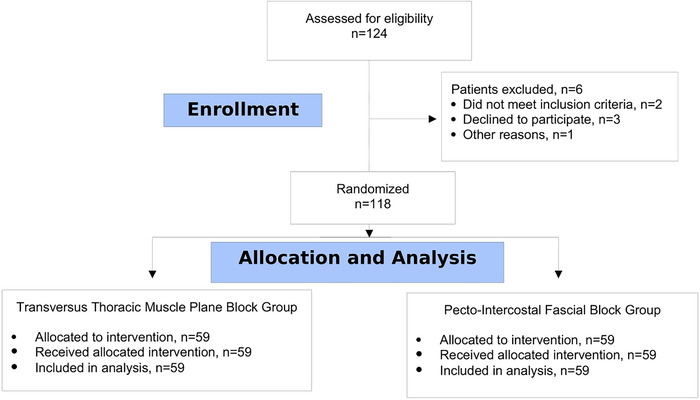
Consolidated Standards of Reporting Trials flow diagram.

One hundred twenty-four patients were initially enrolled for the study; however, 6 were excluded for various reasons as shown in the Consolidated Standards of Reporting Trials flow diagram (Figure 3). The 2 groups were comparable with respect to age, weight, height, and American Society of Anesthesiologists Physical Status. The 2 groups were also similar in terms of diagnosis, duration of mechanical ventilation, and intraoperative fentanyl administration (Table 1).

**Table 1.** Demographic Data by Study Group

Variable	TTMPB Group, n=59	PIFB Group, n=59	*P* Value
Age, years, mean ± SD	52.76 ± 9.41	55.87 ± 8.33	0.30
Weight, kg, mean ± SD	59.88 ± 9.61	62.73 ± 11.39	0.73
Height, cm, mean ± SD	168.45 ± 6.04	170.73 ± 7.12	0.65
American Society of Anesthesiologists Physical Status, n (%)			0.77
II	31 (52.5)	33 (55.9)	
III	28 (47.5)	26 (44.1)	
Diagnosis, n (%)			0.73
Coronary artery disease	34 (57.6)	36 (61.0)	
Valvular heart disease	25 (42.4)	23 (39.0)	
Duration of mechanical ventilation hours, mean ± SD	6.8 ± 1.49	7.0 ± 1.35	0.76
Intraoperative fentanyl administration, μg, mean ± SD	304.3 ± 47.05	311.8 ± 58.55	0.67

PIFB, pecto-intercostal fascial block; TTMPB, transversus thoracic muscle plane block.

**Figure 4.**
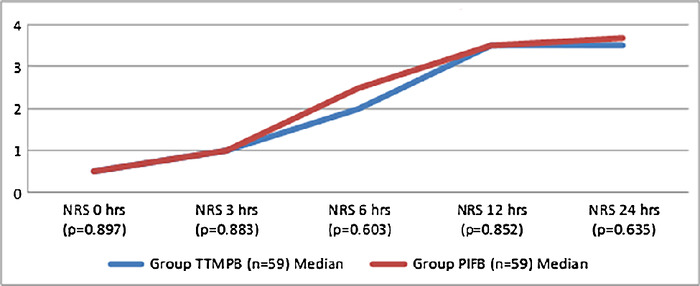
**Median numeric rating scale (NRS) scores obtained during normal tidal volume breathing. The NRS is scored from 0 to 10, with 0 being no pain and 10 being the worst possible pain.** hrs, hours; PIFB, pecto-intercostal fascial block; TTMPB, transversus thoracic muscle plane block.

**Figure 5.**
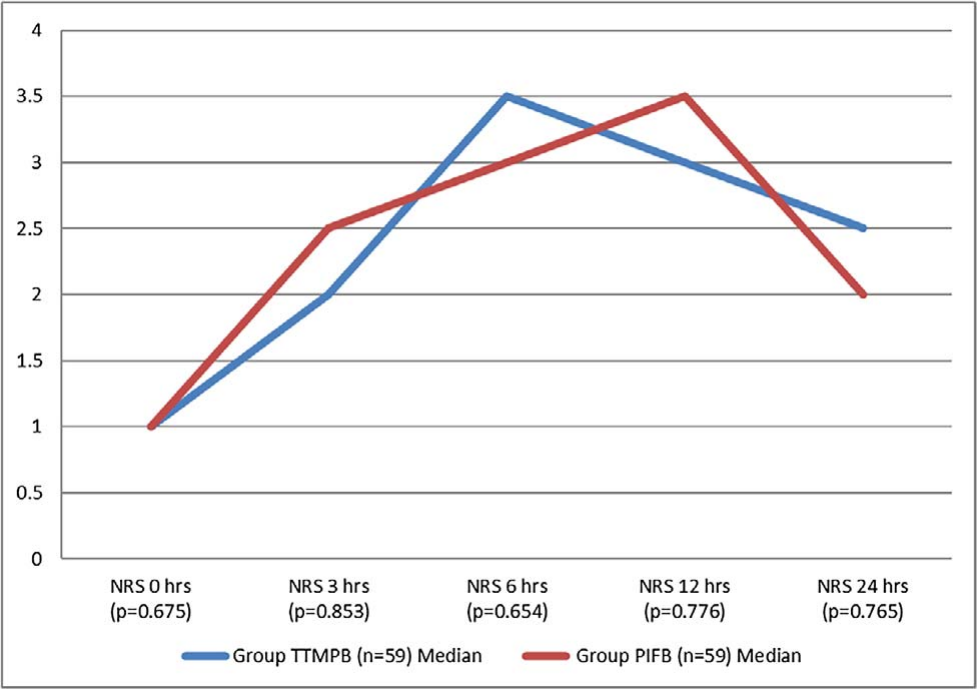
**Median numeric rating scale (NRS) scores obtained during coughing. The NRS is scored from 0 to 10, with 0 being no pain and 10 being the worst possible pain.** hrs, hours; PIFB, pecto-intercostal fascial block; TTMPB, transversus thoracic muscle plane block.

Opioid (tramadol) consumption in the 2 groups was similar (*P*=0.672) (Table 2). Pain scores assessed with the NRS at the 0-, 3-, 6-, 12- and 24-hour intervals during normal tidal volume breathing and during coughing were similar in both the groups (*P*>0.05) (Figures 4 and 5). Time to first rescue analgesia (*P*=0.786), time to extubation (*P*=0.608), and length of ICU stay (*P*=0.598) were similar in the 2 groups. Postoperative complications were vomiting, nausea, and retching, but no significant differences were found between the 2 groups.

**Table 2.** Outcomes by Study Group

Outcome	TTMPB Group, n=59	PIFB Group, n=59	*P* Value
Opioid (tramadol) consumption, mg, mean ± SD	188.65 ± 42.56	194.75 ± 35	0.672
Time to first request for rescue analgesia, hours, median [IQR]	3.5 [1.72, 4.28]	3.75 [1.68, 4.86]	0.786
Time to extubation, hours, median [IQR]	5 [4, 6]	6 [5, 6]	0.608
Length of ICU stay, hours, median [IQR]	45 [35, 49]	39 [34, 47]	0.598
Postoperative complication, n (%)			
Vomiting	1 (1.7)	2 (3.4)	0.558
Nausea	4 (6.8)	3 (5.1)	0.696
Retching	5 (8.5)	4 (6.8)	0.728

ICU, intensive care unit; IQR, interquartile range; PIFB, pecto-intercostal fascial block; TTMPB, transversus thoracic muscle plane block.

## DISCUSSION

Our study results show comparable opioid consumption during the first 24 hours between the 2 groups, and these findings align with a pilot study by Kaya et al,^13^ although Kaya et al compared morphine usage, while our study compared tramadol. A study by Mansour et al found that overall morphine usage in the first 24 hours was significantly lower in the PIFB group vs the TTMPB group, possibly the result of injecting the local anesthetic into the plane at 2 locations (over the second and fourth ribs).^12^ In our study, we injected the local anesthetic at a single location, potentially resulting in better drug spread and analgesic coverage. In our population, pain scores on the NRS at various time points were similar in both groups, consistent with the findings of Kaya et al.^13^ Two studies that compared patients who received these blocks with control groups reported significantly reduced opioid usage in the block groups.^14,15^

The time to first request for rescue analgesia was similar in both our study groups unlike in the Mansour et al study where the time was significantly longer in the PIFB group vs the TTMPB group.^12^ This result could again be attributable to injection of the local anesthetic at 2 locations.

The PIFB has potential benefits as an alternative to the TTMPB. When performing a TTMPB, only a thin layer of transversus thoracic muscle separates the pleura, increasing the risk of puncturing the pleura and causing pneumothorax. On the other hand, with the PIFB, a 3-muscle layer is above the pleura, providing better protection.^12^ In addition, the internal mammary artery lies in the plane where the TTMPB is performed, so the block is associated with a high risk of vascular laceration.^12^ Further, in coronary artery bypass graft surgery that involves harvesting of the internal mammary artery, the spread of local anesthetic may be affected because harvesting the internal mammary artery will disturb the transversus thoracic muscle plane, thereby resulting in an inadequate TTMPB block.^16^ Finally, the intercostal space is known for high systemic absorption and therefore has a high risk for systemic toxicity of local anesthetic with the TTMPB.^11^

In addition to the median sternotomy, other major sources of pain following cardiac surgery are chest tubes and saphenous vein harvesting,^1^ both of which contribute to substantial postsurgical discomfort. These factors likely explain why patients in our study reported pain and sought medication for relief. Our study focused on assessing poststernotomy pain using pain scores, although these scores may not fully capture the extent of the discomfort experienced by patients or their need for pain management. Additionally, local anesthetic infiltration at the site of the chest tube could enhance pain control by offering targeted relief, potentially leading to lower pain scores and improved comfort. However, our study lacked a control group, limiting the ability to make definitive comparisons. Future research would be strengthened by the inclusion of a control group and could provide a more comprehensive understanding of pain management strategies and their effectiveness.

Because of the limited availability of accessible clinical studies, we faced several challenges in making meaningful comparisons with our research. These challenges included variations in study designs, differences in patient populations, the absence of control groups, inconsistent pain measurement methods, and a lack of standardization in the administration of analgesic interventions. Furthermore, the absence of comparative studies of both the TTMPB and PIFB blocks made it difficult to draw direct conclusions. Despite these limitations, our findings support the efficacy of both the TTMPB and PIFB blocks in reducing pain and opioid consumption following median sternotomy.

## CONCLUSION

In managing acute pain after sternotomy, both the TTMPB and the PIFB were associated with similar opioid consumption by patients and similar time to first rescue analgesia request. However, the study did not provide direct evidence to conclude that one technique was more effective than the other. The PIFB may offer a practical alternative to the TTMPB because of ease of administration and favorable anatomic considerations. Although further research is necessary to definitively compare these techniques, the findings of this study suggest that both blocks could be useful options for pain management following median sternotomy. Additional controlled studies are needed to refine the understanding of their comparative effectiveness and guide clinical practice in pain management.

## Notes

*This article meets the Accreditation Council for Graduate Medical Education and the American Board of Medical Specialties Maintenance of Certification competencies for Patient Care and Medical Knowledge.*
